# Where’s the Melancholy?

**Published:** 1889-10

**Authors:** 


### WHERE'S THE MELANCHOLY?

(MACHINE-MADE, FOR THE "RED BACK JEWEL.")

"The melancholy days are come," (says Bryant,
But he lived in Connecticut,) "the saddest
Of the year;" but just where the sad comes in
To yours truly is, just now, not perceptible,—
Can't see it.

With its balmy air and zephyr-breezes,—
("Wailing winds and naked trees" nothing)
The sense of hush,—the gorgeous trees-es—
The purple mist that dims the distance—
October is just splendid!

Why, it's harvest time! See the pumpkins!
And the peas-es! and the pinders! and perhaps—
Potatoes, (or po-turnips), how they tumble in to market!
And the Granger? Is he happy? Rather!
Where's the melancholy?

Purple are the grapes-es now, and juicy
Are the apples; nuts are brown; in the forest
Sings the mocker, (thinks it's spring;)
Red the roses in the garden; and subscriptions
Are just *pouring* in;—where's the sad?

See yon ripple on the water! (imagine the water;)
Why's its solemn stillness thusly broken?
I guess a big old trout has taken
In a fly;—I'll take *him* in!
(Look! beauty, aint he?)

And now the doctor hies him outward; now's the time
He's got to rustle; (not to see a patient
But to get his money;) and with christian charity
Forthwith *he* remembers the poor——
Editor; (where's the spondulicks, doctor?)

Heaped in the hollow of his desk the doctor's bills are laid,
He'll rustle 'mongst his patrons till the last darned one is paid.
The farmer and his men are flush, and in their eyes he sees—
Speculation? No! but *cash*—to pay—in lieu of—**peas!*
Oh, where's the melancholy?

[*For fear that this beautiful point may not be clearly seen by the city physician, it is necessary to explain that too often the poor tired country doctor has to take his "pay in kind"—in "chips and whetstones"—farm products, or anything he can get. During the war, it is said, that in North Carolina herrings were the circulating medium, and not infrequently the store-keeper was requested to put up a herring's worth of sugar. One rustling doctor—a friend of ours, wrote us that he "collected on account" (in one day) a parrot, two canary birds, four bushels of black-eye peas, and a calf, worth four dollars! Lucky Doctor;—"and," he added, "the farmer 'throwed in' a cat!" Fact!]

